# Integrative analysis of key candidate genes and signaling pathways in acute coronary syndrome related to obstructive sleep apnea by bioinformatics

**DOI:** 10.1038/s41598-021-93789-2

**Published:** 2021-07-08

**Authors:** Yanxi Shi, Zhengye Jiang, Liqin Jiang, Jianjiang Xu

**Affiliations:** 1 Department of Cardiology, Jiaxing Second Hospital, Jiaxing, China; 2 Institute of Neurosurgery, School of Medicine, Xiamen University, Xiamen, China

**Keywords:** Computational platforms and environments, Data mining, Data processing

## Abstract

Although obstructive sleep apnea (OSA) has been clinically reported to be associated with acute coronary syndrome (ACS), the pathogenesis between the two is unclear. Herein, we analyzed and screened out the prospective molecular marker. To explore the candidate genes, as well as signaling cascades involved in ACS related to OSA, we extracted the integrated differentially expressed genes (DEGs) from the intersection of genes from the Gene Expression Omnibus (GEO) cohorts and text mining, followed by enrichment of the matching cell signal cascade through DAVID analysis. Moreover, the MCODE of Cytoscape software was employed to uncover the protein–protein interaction (PPI) network and the matching hub gene. A total of 17 and 56 integrated human DEGs in unstable angina (UA) and myocardial infarction (MI) group associated with OSAs that met the criteria of |log2 fold change (FC)|≥ 1, adjusted *P* < 0.05, respectively, were uncovered. After PPI network construction, the top five hub genes associated with UA were extracted, including APP, MAPK3, MMP9, CD40 and CD40LG, whereas those associated with MI were PPARG, MAPK1, MMP9, AGT, and TGFB1. The establishment of the aforementioned candidate key genes, as well as the enriched signaling cascades, provides promising molecular marker for OSA-related ACS, which will to provide a certain predictive value for the occurrence of ACS in OSA patients in the future.

## Introduction

Obstructive sleep apnea (OSA) represents a severely underdiagnosed (~ 80%) form of sleep-disordered breathing^1–6^. Obstructive sleep apnea (OSA) is highly prevalent in patients with cardiovascular diseases^7,8^. Increasing evidence indicates that OSA is associated with incidence and progression of coronary artery disease^9–11^ and cerebrovascular disease^12^. Compared with the general population, prevalence of OSA is higher in acute coronary syndrome (ACS) patients and ranges from 36 to 63% across various ethnicities^13^. Notably, among patients with coronary artery disease, those with ACS represent a high-risk subset and generally have higher mortality than patients with stable angina^14^. In addition, observational studies have examined whether OSA significantly increased the risk of recurrent cardiovascular events in patients with ACS and/or undergoing percutaneous coronary intervention (PCI)^15–18^. Despite the huge advancements in ACS research, the prognosis of ACS treatment is still poor. With the onset age of ACS patients gradually getting younger, it is imperative to establish the etiology, as well as the molecular features, of ACS disease. Therefore, we explore the molecular biomarkers by studying the correlation between OSA and ACS disease to provide evidence for early diagnosis, prevention, and treatment of this disease.

At present, high-throughput sequencing techniques such as molecular diagnosis, prognosis estimation, as well as drug target discovery (which can be employed to assess the gene expression differences, as well as the variable splicing variation), are gradually considered to have important clinical significance in disease research. The Integrated Gene Expression Database (GEO), a publicly available website supported by the National Center for Biotechnology Information (NCBI), harbors dozens of basic experimental disease gene expression patterns and is extensively employed to explore key genes and prospective mechanisms of disease onset and development^19^. Though the pathogenesis of OSA has been found to be related to ACS recently, its pathogenesis, as well as the molecular mechanism remain unknown. Hence, we need to utilize the gene expression chip in the bulletin database and analyze its data through modern software to find new diagnostic markers and therapeutic targets^20^.

In this study, we retrieved human unstable angina (UA) and myocardial infarction (MI) gene expression patterns GSE60993 and GSE24519 from the GEO website. After that, the R software (version 3.6.3) was used to screen for differentially expressed genes (DEG)^21,22^. Then, the genes related to obstructive sleep apnea were obtained by text mining. We use the online tool Venny to analyze the intersection of DEG and text mining gene sets to obtain common genes, and we further used different bioinformatics methods to perform gene ontology, signal pathway enrichment annotations, protein and protein interactions for these common genes the study. In this article, we have studied candidate genes and signal pathways involved in ACS related to OSA, which will help provide a certain predictive value for the occurrence of ACS in OSA patients in the future.

## Methods

### Data abstraction

We abstracted the gene expression chip data GSE60993 and GSE24519 from the NCBI Gene Expression Comprehensive (GEO) web resource (https://www.ncbi.nlm.nih.gov/geo/)^19,23^. The GSE60993 cohort contains seven normal control and nine UA samples, while the GSE24519 dataset includes four normal control and four MI samples.

#### Identification of DEGs

The core R package was used to process the downloaded matrix files. After normalization, the differences between UA or MI and the control group were determined by truncation criteria |log2 fold change (FC)|≥ 1, adjusted *P* < 0.05), and we selected the remarkable DEGs for downstream analyses^24^. In order to further investigate DEGs, we selected 10 up-regulated and 10 down-regulated mRNAs with the most fold change as the candidate DEGs for subsequent studies (Table 1).

**Table 1:** Top 10 up-regulated and down-regulated DEGs.

UA				MI			
Gene name	Expression	Log2FC	P value	Gene name	Expression	Log2FC	P value
LIN37	Up	1.433057	0.000275	KRI1	Up	36.88214	1.90E−10
PGLYRP1	Up	5.610778	0.000294	ATXN7L1	Up	42.96463	2.60E−10
MYL6	Up	1.58533	0.000461	PURA	Up	35.266	7.85E−09
RASSF2	Up	1.710884	0.000729	C2orf48	Up	32.62867	2.92E−06
FAM49A	Up	1.129013	0.000729	SLC35A4	Up	12.59876	1.23E−05
DOK4	Up	1.259271	0.000773	LOC101928651	Up	9.989054	2.97E−05
LPIN2	Up	1.28557	0.001009	BPTF	Up	7.618646	4.80E−05
RTN3	Up	2.057976	0.001314	MTMR2	Up	6.380524	5.20E−05
GCAT	Up	1.740766	0.0014	NDC1	Up	12.41982	6.80E−05
LRRFIP1	Down	−1.60294	HPCAL1	LALBA	Down	−21.5901	5.31E−09
HPCAL1	Down	−1.08925	SLC39A9	TBX19	Down	−21.956	6.07E−09
SLC39A9	Down	−1.05401	ARSD	STS	Down	−11.8866	5.73E−07
ARSD	Down	−4.44053	JCHAIN	F10	Down	−14.2408	8.61E−07
JCHAIN	Down	−1.94655	CMPK2	SSX3	Down	−13.1658	8.75E−07
CMPK2	Down	−1.28788	RAD51C	RAB11FIP4	Down	−11.122	1.48E−06
RAD51C	Down	−1.22599	MS4A1	GPRC5C	Down	−13.2281	2.75E−06
MS4A1	Down	−1.4018	FASTKD2	SRI	Down	−17.7746	3.71E−06
FASTKD2	Down	−1.45227	GCH1	LDB2	Down	−9.68058	3.92E−06
GCH1	Down	−1.50271	OAS2	P3H2	Down	−17.1589	5.15E−06
OAS2	Down	−1.60294	HPCAL1	LALBA	Down	−21.5901	5.31E−09

*DEGs* differentially expressed genes, *Log2FC* log2 fold-change, *UA* unstable angina, *MI* myocardial infarction.

#### Text mining

We carried out the text mining based on the pubmed2ensembl public tool (http://pubmed2ensembl.ls.manchester.ac.uk/). When manipulated, pubmed2ensembl retrieves all the gene names found in the existing literature relevant to the search topic. We searched for the concept of “obstructive sleep apnea”. We then screened all the genes associated with the topic from the results. Finally, we used the gene set obtained by text mining and the previously obtained differential gene set for the next step of analysis after the intersection.

#### Gene ontology analysis of DEGs and KEGG pathway analysis

The obtained DEGs were imported to David V. 6.8 (https://david.ncifcrf.gov/). The GO annotation and KEGG pathway enrichment were carried out using the web resource^25–27^, which provided a sequence of functional annotation tools for systematic analysis of biological significance of gene lists. The above gene tables were analyzed with *P* < 0.05 as the significant threshold.

#### Assessment of the PPI network of the DEGs

We used the STRING online search tool to analyze the protein–protein interaction (PPI) data encoded by DEG^28^, and only the combination score > 0.6 was considered significant. Then, the PPI network was analyzed and visualized by using Cytoscape, and the first five hub genes were determined as per the connectivity between DEGs. The standard default setting of the MCODE parameter. The function enrichment of DEGs of each module was analyzed, with *P* < 0.05 as the cutoff standard.

#### Diagnostic analysis of DEGs and prognosis.

In order to further analyze whether the differentially expressed target mRNA related to prognosis has diagnostic value, subject receiver operating characteristic curve (ROC) analysis uses the open-source network tool Hiplot (https://hiplot.com.cn/basic).

## Results

### DEGs identification

**Figure 1:**
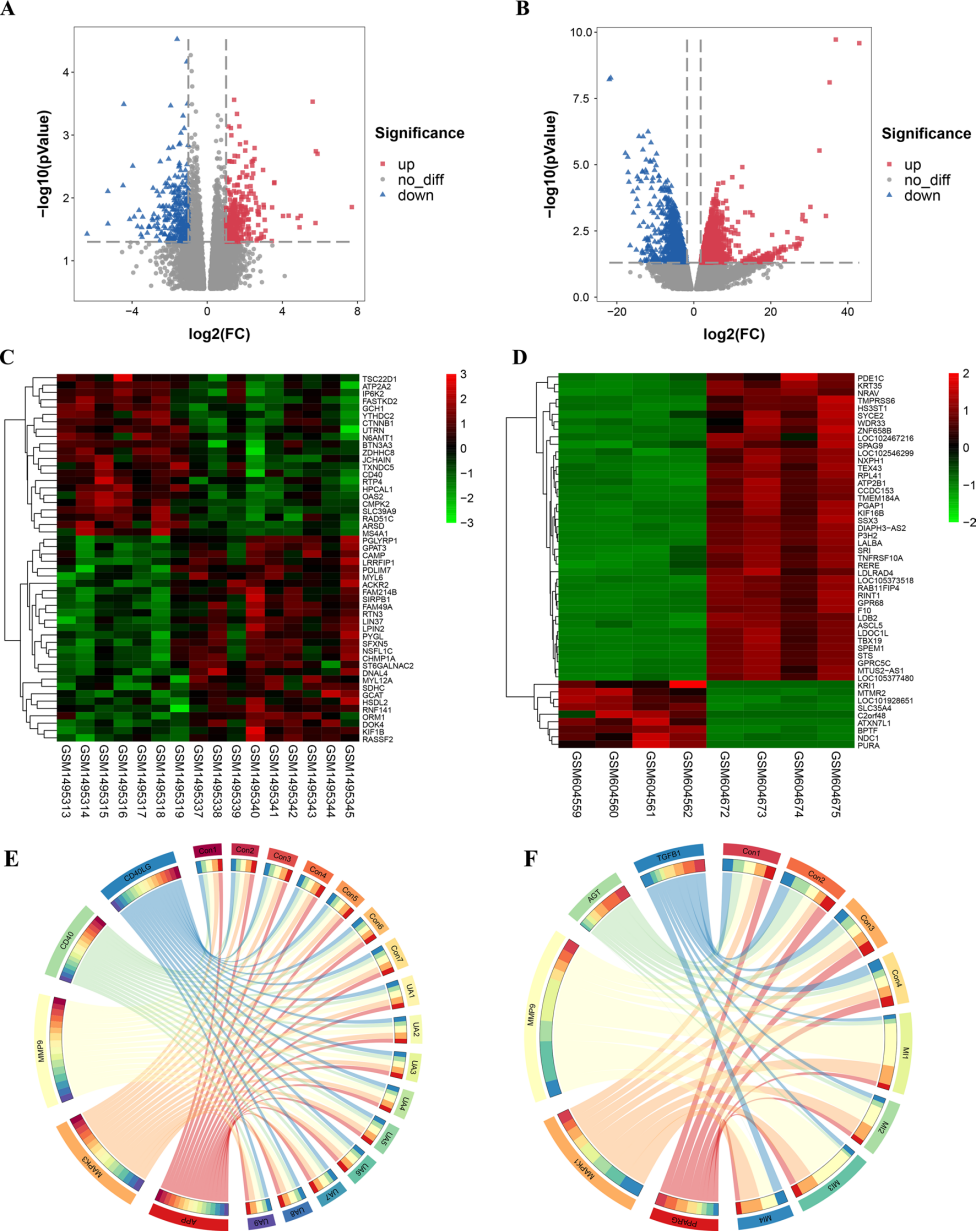
Differentially expressed genes between unstable angina/myocardial infarction and control groups. (**A,C)** Volcano plot and cluster heat map of the top 50 differentially expressed genes from GSE60993. (**B,D)** Volcano plot and cluster heat map of the top 20 differentially expressed genes from GSE24519. (**E,F)** Screened the gene correlation maps between the top 5 genes of UA and MI and their corresponding samples. Red represents the upregulated genes based on |log2FC|> 1 and P value < 0.05 and blue represents the downregulated genes based on the same statistical requirements.

Firstly, 587 DEGs were selected from UA samples and normal controls in the GSE60993 data set through limma package screening of R software. Of these, 299 upregulated genes and 288 downregulated genes were selected. At the same time, 2916 differentially expressed genes, including 1647 upregulated genes and 1269 downregulated genes, were obtained by analyzing the MI samples in the GSE24519 data set and the normal control group. Then, the overall distribution of the two data sets and the first 100 DEGs are represented by volcano map and heatmap, respectively (Fig. 1A–D). Figure 1E,F, respectively, show the correlation between the top 5 differential genes in UA and MI in each sample. Sample using |log2 fold change (FC)|≥ 1 criteria and adjusted *P* < 0.05.

**Figure 2:**
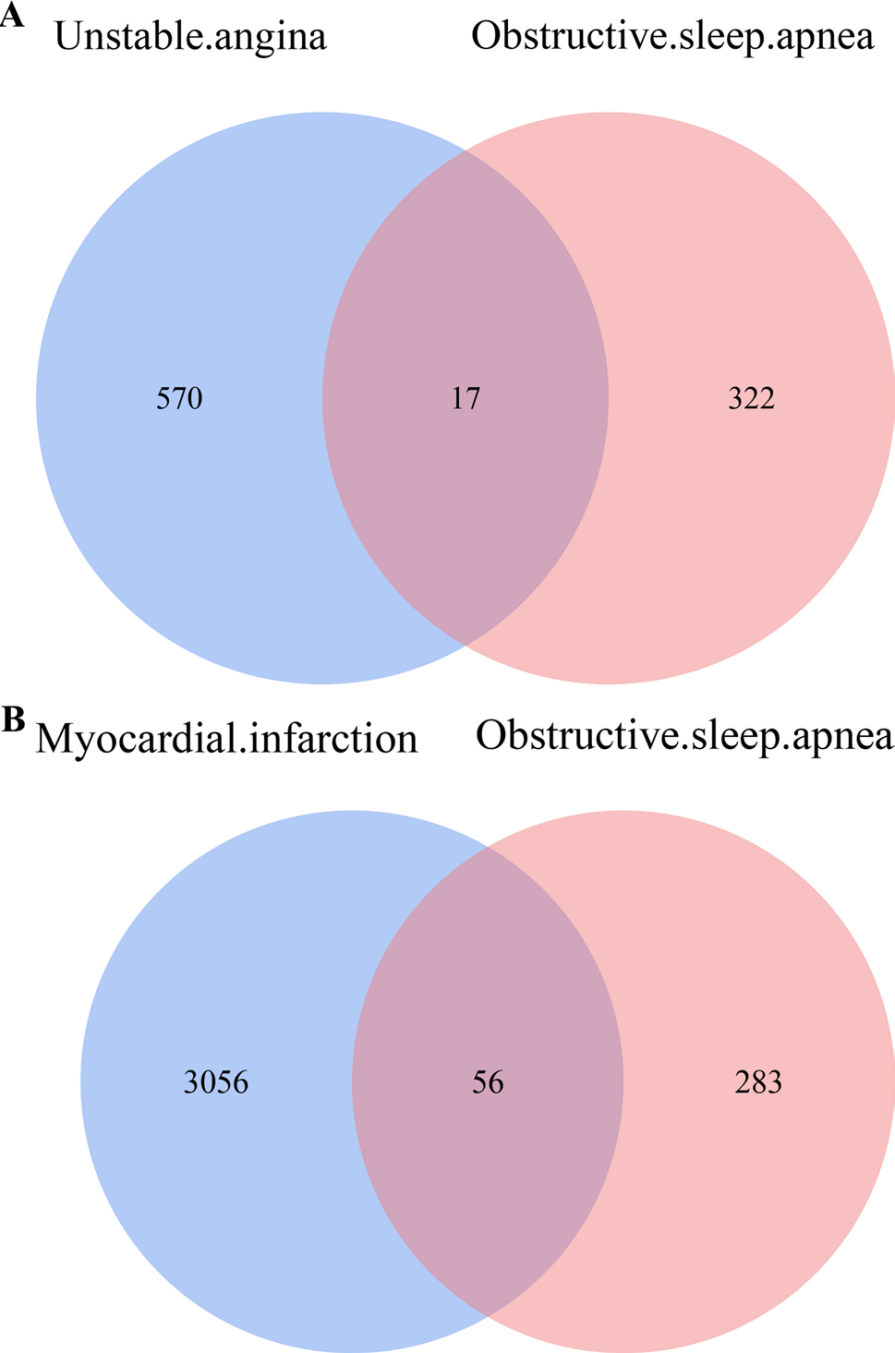
Venn diagram of DEGs from microarray data and genes list from text mining. (**A)** Intersection of genes between DEGs generated from GSE60993 and obstructive sleep apnea gene list from text mining. (**B)** Intersection of genes between DEGs generated from GSE24519 and obstructive sleep apnea gene list from text mining. DEGs, differentially expressed genes.

Through text mining, 339 human genes associated with OSA were selected. After the DEGs in the microarray data were crossed, the intersection of selected genes was obtained, and 17 genes involved in UA group and 56 genes involved in MI group were obtained (Fig. 2A,B).

#### Function and signal pathway enrichment analysis

**Figure 3:**
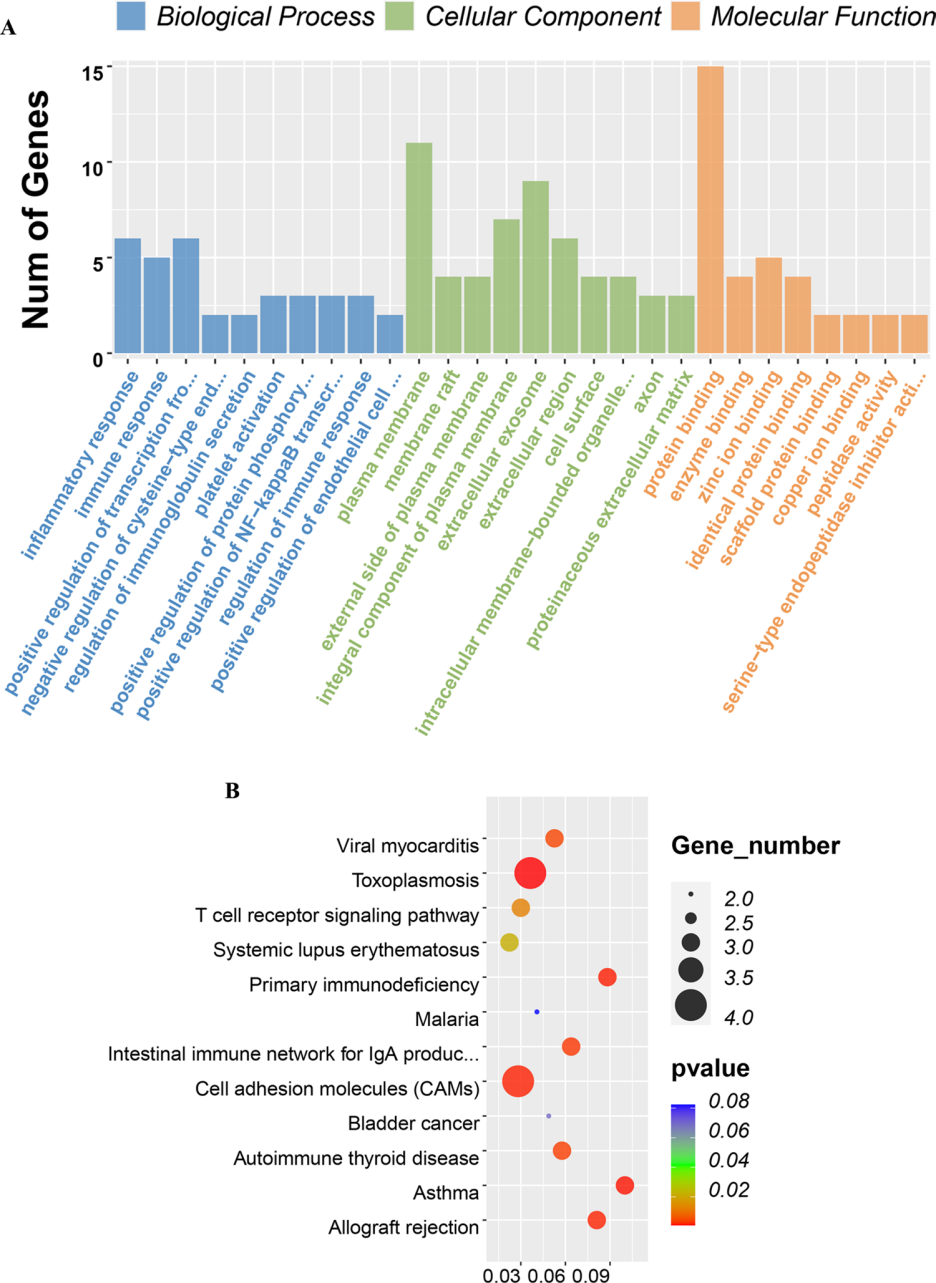
GO term and KEGG pathway analysis for DEGs significantly associated with unstable angina and obstructive sleep apnea. (**A)** Top 10 GO terms. Number of gene of GO analysis was acquired from DAVID functional annotation tool. p < 0.05. (**B)** KEGG pathway.

**Figure 4:**
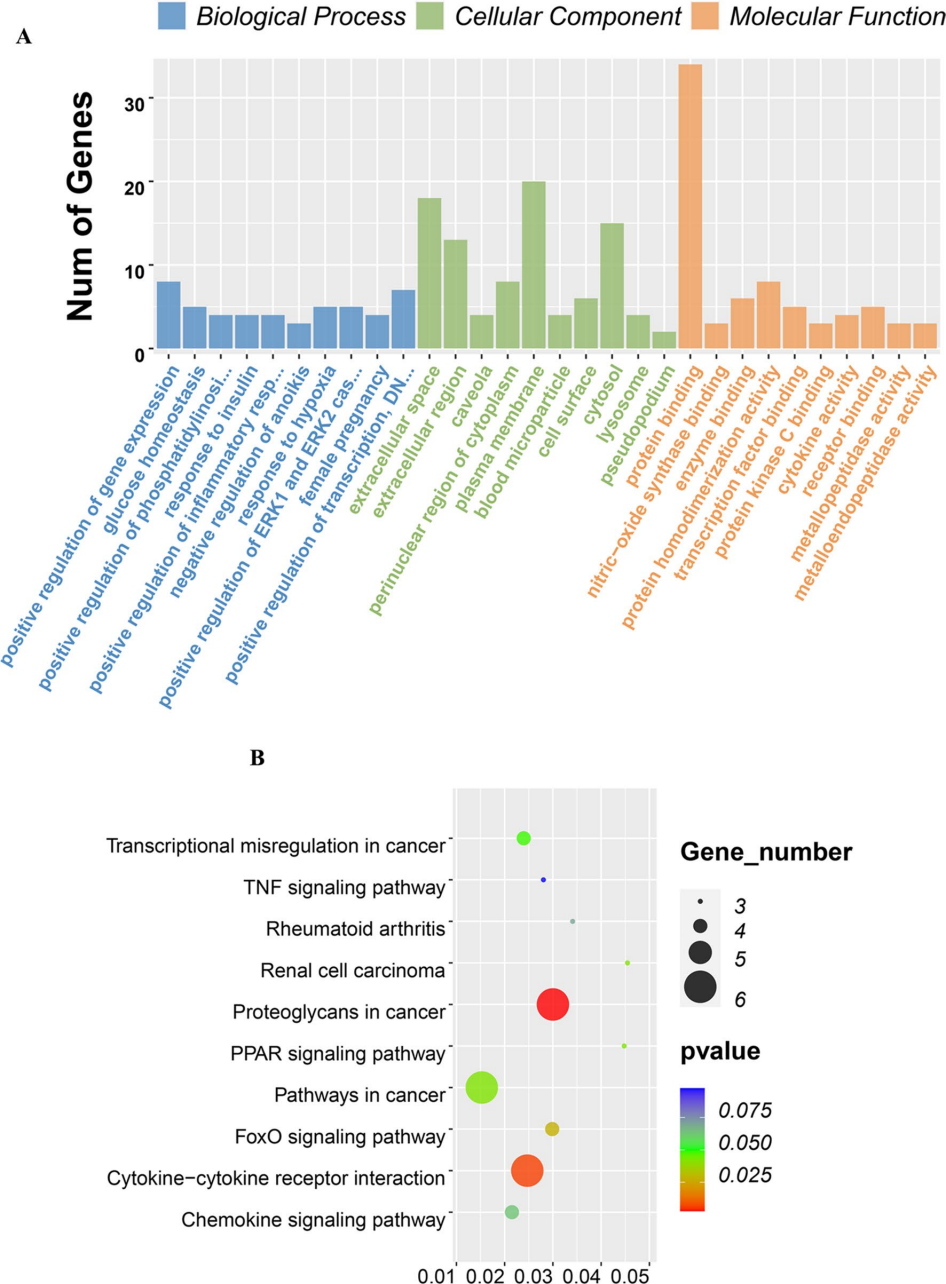
GO term and KEGG pathway analysis for DEGs significantly associated with myocardial infarction and obstructive sleep apnea. (**A)** Top 10 GO terms. Number of gene of GO analysis was acquired from DAVID functional annotation tool. p < 0.05. (**B)** KEGG pathway.

After introducing the DEGs obtained above into DAVID, we subjected them to GO and KEGG enrichment analysis to study the biological functions of DEGs integrated in UA and MI associated with chronic periodontitis. In the GO analysis results, 27 biological process terms (BP), 15 cell component terms (CC), and 8 molecular function terms (MF) were uncovered in the DEGs integrated by UA. *P* < 0.05 signified threshold significance. Overall, 6 genes were primarily abundant in BP term to “inflammatory response”, 11 genes were located in the “plasma membrane” of CC term, and 15 genes were abundant in the MF term “protein binding” as indicated in Fig. 3A. For MI, integrated DEGs were remarkably abundant in 139 GO terms consisting of 103 BP terms, 18 CC terms and 18 MF terms. Besides, the genes were abundant in the following terms: modulation of positive regulation of gene expression in BP and extracellular space in CC, as well as protein binding in MF, which constituted the top 3 GO annotation terms in which the integrated genes were most remarkably enriched (Fig. 4A).

The KEGG enrichment assessment demonstrated that the integrated DEGs were remarkably enriched in the KEGG cascade Toxoplasmosis, Asthma and Primary immunodeficiency in UA group (Fig. 3B) and Proteoglycans in cancer, Cytokine-cytokine receptor interaction and FoxO signaling pathway in the MI group (Fig. 4B).

#### Module screening from the PPI network

**Figure 5:**
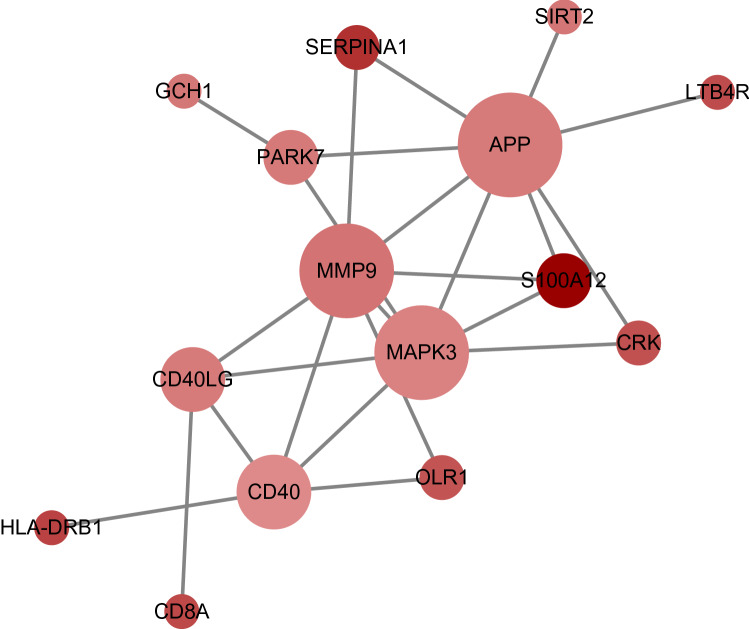
Based on the STRING online database, 17 genes/node were filtered into the DEG PPI network. The color of a node in the PPI network reflects the log2 (FC) value of the Z score of gene expression, and the size of node indicates the number of interacting proteins with the designated protein.

**Figure 6:**
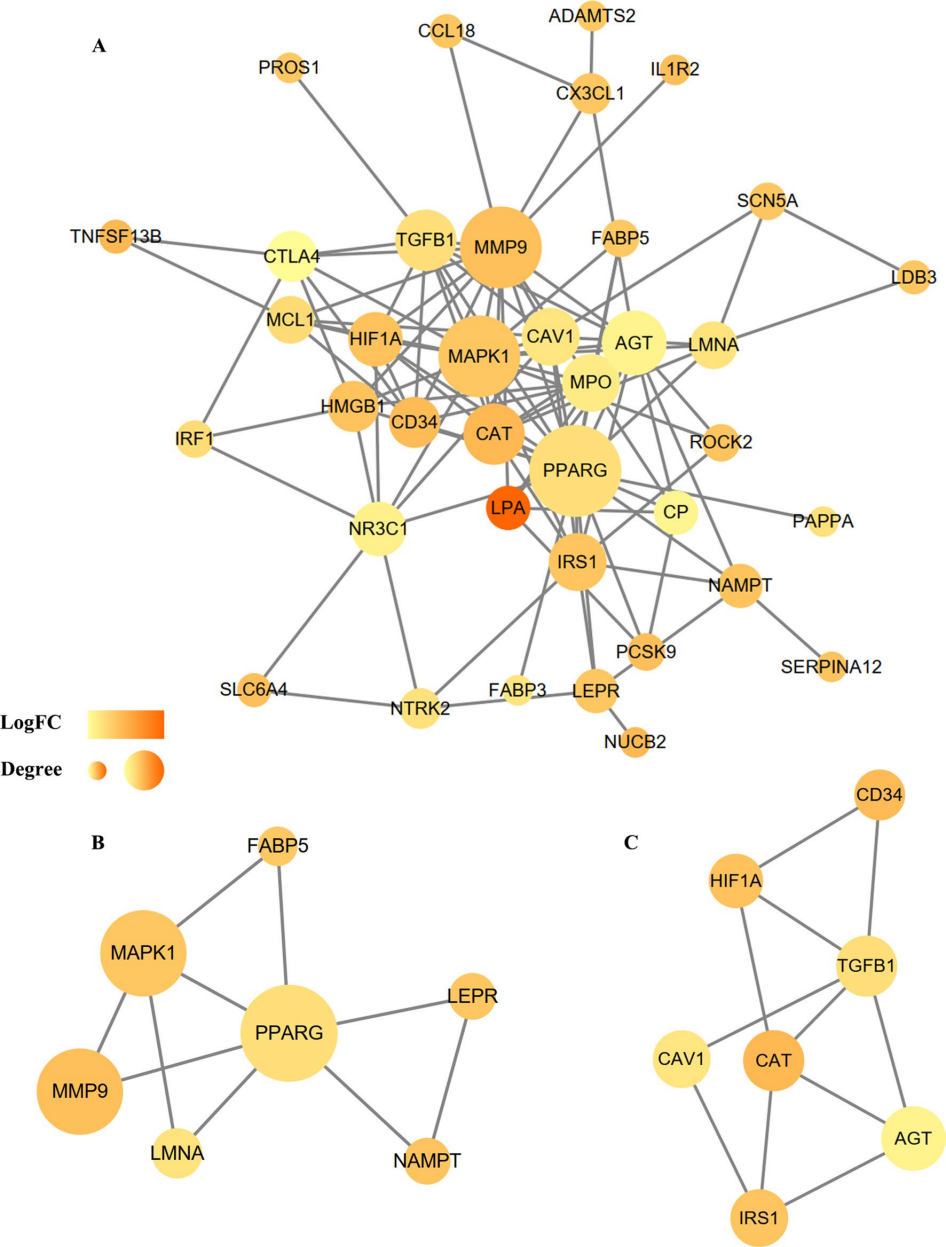
**(A)** Based on the STRING online database, 56 genes/node were filtered into the DEG PPI network. (**B)** The most significant module 1 from the PPI network. (**C)** The second significant module 2 from the PPI network. The color of a node in the PPI network reflects the log2 (FC) value of the Z score of gene expression, and the size of node indicates the number of interacting proteins with the designated protein.

Based on the 17 UA group genes and the 56 MI group genes, the Cytoscape publicly available platform and the STRING resource were employed to develop the PPI network, perform module analysis, and visualization. Consequently, we developed a PPI network bearing 24 crosstalk based on 15 integrated DEGs related to UA (Fig. 5). Moreover, we developed a PPI network in the MI group containing 38 integrated DEGs (Fig. 6A). Based on the degree value, the top five hub genes extracted from the UA group consisted of APP (amyloid beta precursor protein), MAPK3 (mitogen-activated protein kinase 3), MMP9 (matrix metallopeptidase 9), CD40 (CD40 molecule) and CD40LG (CD40 ligand). On the other hand, in the MI group, the top five hub genes were PPARG (peroxisome proliferator activated receptor gamma), MAPK1 (mitogen-activated protein kinase 1), MMP9 (matrix metallopeptidase 9), AGT (angiotensinogen), and TGFB1 (transforming growth factor beta 1) (Table 2).

**Table 2:** Top five hub genes identified from the PPI networks.

Unstable angina related genes		Myocardial infarction related genes	
Gene	Node	Gene	Node
APP	16	PPARG	38
MAPK3	14	MAPK1	32
MMP9	14	MMP9	32
CD40	10	AGT	22
CD40LG	8	TGFB1	20

We employed the MCODE algorithm to determine highly interconnected subnets, which are frequently protein complexes, as well as components of cascades, as per the topological structure. However, we found that there is no highly clustered mou module in UA by calculation. So, we selected the two most important modules from MI group for further analysis (Fig. 6B,C). Additional functional enrichment assessment of the established modules demonstrated that genes in the MI module were abundant in the GO terms of “glucose homeostasis”, “caveola”, “enzyme binding”, and the KEGG cascade of “FoxO signaling pathway” (Table 3).

**Table 3:** The significant gene ontology terms of the established modules.

Term	Category	Description	Count	*P*-value
GO:0042593	BP	Glucose homeostasis	4	5.78E−05
GO:0001666	BP	Response to hypoxia	4	2.80E−04
GO:0045893	BP	Positive regulation of transcription, DNA-templated	5	5.01E−04
GO:0005901	CC	Caveola	3	9.53E−04
GO:0005829	CC	Cytosol	8	0.004018
GO:0048471	CC	Perinuclear region of cytoplasm	4	0.008723
GO:0019899	MF	Enzyme binding	5	9.24E−05
GO:0008134	MF	Transcription factor binding	4	0.001189
GO:0005515	MF	Protein binding	12	0.016236
hsa04068	KEGG	FoxO signaling pathway	4	0.001792
hsa05200	KEGG	Pathways in cancer	5	0.004964
hsa04152	KEGG	AMPK signaling pathway	3	0.021747

*GO* gene ontology, *BP* biological processes, *CC* cellular composition, *MF* molecular function, *KEGG* Kyoto encyclopedia of genes and genomes.

#### Diagnosis prediction of DEGs related to prognosis.

**Figure 7:**
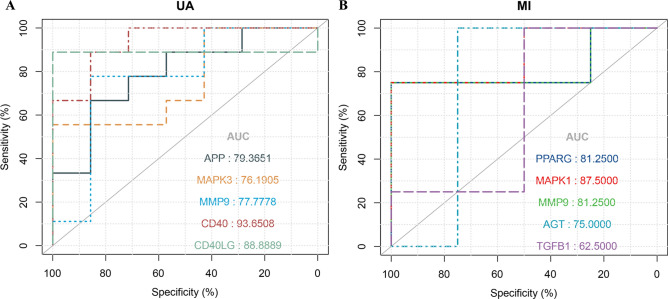
**(A)** ROC curves of APP, MAPK3, MMP9, CD40 and CD40LG expression in unstable angina. (**B)** ROC curves of PPARG, MAPK1, MMP9, AGT, and TGFB1 expression in myocardial infarction.

We used the ROC curve to research the prediction effects of APP, MAPK3, MMP9, CD40 and CD40LG in UA, and PPARG, MAPK1, MMP9, AGT and TGFB1 in MI (Fig. 7). Interestingly, the AUC values of the above-mentioned differentially expressed target mRNAs are all > 0.7. This result suggests that for OSA patients, the above genes have potential diagnostic value for UA and MI.

### Ethical approval

This article does not contain any studies with human participants or animals performed by any of the authors.

### Consent for publication

All authors consent to the publication of this study.

## Discussion

In a multicenter international study, OSA was shown to independently predict adverse cardiovascular events. Therefore, a new potential treatment method for preventing the progression of ACS has emerged: active treatment OSA. However, at present, the pathogenesis and effective treatment of OSA for ACS remain unclear. Hence, it is imperative to explore the molecular mechanism of the ACS after OCS to determine efficient biomarkers and effective approaches for the diagnosis, monitoring, and treatment of patients. In previous reports, new genetic variations and associated quantitative traits associated with OSA susceptibility have been identified at the genome-wide significance level. In previous reports, new genetic variations and associated quantitative traits associated with OSA susceptibility have been identified at the genome-wide significance level^29^. Meanwhile, Farias et al. recently have discovered that that allele rs12415421 was an important predictor of the incidence of OSA^30^.

Herein, 17 genes in UA and 56 genes in MI linked to OSA were uncovered for functional analysis using the GO, and the KEGG enrichment assessments. Additionally, the PPARG gene comprised one of the hub genes uncovered by the PPI network. PPARG can adjust the balance between glucose and fatty acid oxidation, which plays an important role in the reconstruction of human myocardial infarction after ischemia^31–33^. Moreover, previous evidence has suggested that PPARG may be a risk factor for cardiovascular diseases such as metabolic syndrome, obesity, diabetes and hypertension^34–37^. PPARG is a member of the nuclear hormone receptor superfamily, which can the recruit the transcriptional coactivators necessary to initiate the transcription of target genes and may also play a protective role in the development of MI in many studies^38–39^. At the same time, previous studies have also found that PPARG may play an important role in the pathophysiological mechanism of OSA^40,41^, and qPCR detection suggests that the expression of PPARG in OSA patients is significantly different from that in the control group^42^.

The functional enrichment analysis of the highly connected module showed that the genes in the MI module were mainly enriched in the KEGG term FoxO signaling pathway. FoxOs participate in a variety of cell functions including apoptosis, cell differentiation, desoxyribonucleic acid injury and repair^43,44^. FoxOs are key signal proteins within the signal transduction of growth factors and are also regulated by the ubiquitin proteasome pathway. In previous studies, the FoxO regulatory pathway had been triggered by the PI3K/AKT pathway, which mediates the function of cell proliferation and growth^45^. So far, the correlation between FoxO signaling pathway and OSA and ACS has not been reported in the previous literature.

The cleaved product of the glycoprotein amyloid precursor protein (APP) is Aβ, which aggregates into Aβ plaques. According to the amyloid cascade hypothesis, it is these plaques that are responsible for AD pathology^46^. Soluble Aβ species can bind to and produce toxicity to various neuronal receptors, leading to cellular oxidative stress and epigenetic-mediated transcription disorders^47^. However, recent studies have shown that soluble Aβ has beneficial physiological effects on certain functions, such as regulating cellular signaling pathways and synaptic function^48^. The main driving force of the pathological progression of AD is the accumulation of Aβ in the brain, which leads to synaptic loss and neuronal cell death^49–51^. In addition, some evidence has been found that the continuous accumulation of cerebrovascular Aβ plays a role in cerebral microhemorrhage ^52,53^ and vascular cognitive impairment^54^.

CD40 is a costimulatory molecule in the constitutive expression of B lymphocytes and is expressed in a variety of cells, such as endothelial cells (ECs), monocytes, macrophages and smooth muscle cells (SMCs)^55^. In Antoniades et al. 's study, CD40 was found to be involved in the immune pathogenesis of ACS ^56^ due to its bi-cellular activation through the signaling pathways C-Jun, NF-κB and ERK 1/2, resulting in the secretion of inflammatory cytokines, adhesion molecules, and platelet activation^55^. However, soluble forms of CD40 and CD40L were significantly associated with adverse cardiovascular events in patients with ACS^57,58^, suggesting that they are potential targets for therapeutic agents ^56^. A recent study found that the soluble CD40 ligand (sCD40L) level of OSA patients is significantly increased, and it can be improved by continuous positive airway pressure therapy^59^. This means that OSA seems to have a clear correlation with the pre-atherosclerotic state, and this state can be continuously monitored by measuring the level of sCD40L.

MMP9 has been shown in many studies to be significantly associated with cardiovascular disease. Moreover, it was also confirmed in our results that MMP9 was highly expressed in both datasets. MMP9 is a protease of the MMP family that is capable of degrading a broad spectrum of extracellular matrix components and is held responsible for vascular remodeling and breakdown of the fibrous cap of atherosclerotic lesions leading to plaque vulnerability^60^. MMPs are a family of zinc-dependent proteinases capable of degrading various structural components of ECM, thus leading to ECM destruction and plaque rupture^61^. At the same time, MMP9 has been found in a number of autoimmune diseases and biological processes, such as pulmonary inflammation, hemorrhage, infection and cancer^62,63^. Therefore, we believe that MMP9 may have a certain relationship with OSA.

MAPK1 is mostly concentrated in the cytoplasm, and activated MAPK1 translocates to the nucleus and activates the expression of target genes in tumor tissues^64^. Many previous studies have demonstrated that MAPK1 plays an important role in atherosclerotic lesions or processes^65–67^. Furthermore, MAPK1 were both up-regulated in coronary heart disease (CAD)^68^. At the same time, MAPK pathway also plays a role in stroke progression^69,70^.

In addition to the genes described above that are known to be associated with OSA and ACS, we also found four potential targeted genes that have not been clearly reported in the literature.

MAPK3, referred to as the mitogen-activated protein kinase 3, is a MAP kinase family member and participates in an extensive array of biological processes, including cell proliferation, as well as angiogenesis. MAPK3 may serve as the intrafollicular mediator that triggers the expansion of the cumulus cell-oocyte complex (COC), as well as the maturation of the oocytes^71–73^. The extracellular, as well as intracellular, mitogenic stimuli activate the MAPK3 cascade, which has pivotal functions in cellular differentiation, proliferation and survival^74^. The study of colorectal cancer by Schmitz et al. showed that the expression of MAPK3 is related to poor prognosis^75^.

Angiotensin (AGT), a plasma globulin of the silk fibroin family, is converted to angiotensin I by renin. Angiotensin converting enzyme (ACE) cleaves angiotensin I and converts to angiotensin II. Angiotensin II then causes increased arterial pressure by participating in intravascular fluid volume elevation and vasoconstriction. Finally, angiotensin II functions through angiotensin receptor type 1 (AGTR1) and angiotensin receptor type 2 (AGTR2)^76–79^.

According to previous reports^80–85^, TGFβ1 is secreted by a variety of cells, such as peripheral blood monocytes, macrophages, platelets, vascular smooth muscle cells (VSMCs), and renal cells. Its regulatory function on the vessel wall is directed at VSMC, endothelial cells and extracellular matrix. Although there is a significant correlation between TGFβ1 and the pathogenesis of atherosclerosis, the relationship between plasma TGFβ1 levels and the risk of ACS remains unclear^81,86–88^. This is because the exact mechanism of TGFβ1 signaling in the vascular system is still not fully understood^82,83,85,89^.

The CD40L gene consists of five exons and four introns. Studies have shown that if CD40L expression is low or it is not expressed, impaired immunoglobulin class-switching while mice overexpressing CD40L have chronic inflammation^90^. Notably, dinucleotide microsatellite with cytosine-adenine (CA) repeats in the CD40LG 3-untranslated region (3-UTR) described as highly polymorphisms have been found to be associated with multiple diseases, such as multiple sclerosis (MS), systemic lupus erythematosus (SLE), and rheumatoid arthritis (RA)^91–93^.

## Conclusions

By employing a sequence of bioinformatics tools for gene expression profiling, we established the core function of candidate key genes, and the enriched signaling cascades constituting the “FoxO signaling pathway” in the molecular modulation network of ACS via integrated bioinformatic analysis. This provides a possible target for the prediction and clinical treatment of ACS in OSA patients in the future. However, in vitro, as well as in vivo studies, should be conducted to verify our findings.

## Data Availability

All data is available under reasonable request.
